# Lipidome and metabolome analyses reveal metabolic alterations associated with MCF-7 apoptosis upon 4-hydroxytamoxifen treatment

**DOI:** 10.1038/s41598-023-45764-2

**Published:** 2023-10-29

**Authors:** Kazuki Nishimoto, Nobuyuki Okahashi, Masaharu Maruyama, Yoshihiro Izumi, Kohta Nakatani, Yuki Ito, Junko Iida, Takeshi Bamba, Fumio Matsuda

**Affiliations:** 1 Department of Bioinformatic Engineering, Graduate School of Information Science and Technology, Osaka University, 1-5 Yamadaoka, Suita, Osaka 565-0871 Japan; 2 Department of Biotechnology, Osaka University Shimadzu Analytical Innovation Research Laboratory, Graduate School of Engineering, Osaka University, 2-1 Yamadaoka, Suita, 565-0871 Japan; 3 Industrial Biotechnology Initiative Division, Institute for Open and Transdisciplinary Research Initiatives, Osaka University, 2-1 Yamadaoka, Suita, 565-0871 Japan; 4 Division of Metabolomics, Medical Research Center for High Depth Omics, Medical Institute of Bioregulation, Kyushu University, 3-1-1 Maidashi, Higashi-ku, Fukuoka, 812-8582 Japan; 5 Analytical and Measuring Instruments Division, Shimadzu Corporation, 1 Nishinokyo Kuwabara-cho, Nakagyo-ku, Kyoto, 604-8511 Japan

**Keywords:** Lipidomics, Metabolomics

## Abstract

4-hydroxytamoxifen (OHT) is an anti-cancer drug that induces apoptosis in breast cancer cells. Although changes in lipid levels and mitochondrial respiration have been observed in OHT-treated cells, the overall mechanisms underlying these metabolic alterations are poorly understood. In this study, time-series metabolomics and lipidomics were used to analyze the changes in metabolic profiles induced by OHT treatment in the MCF-7 human breast cancer cell line. Lipidomic and metabolomic analyses revealed increases in ceramide, diacylglycerol and triacylglycerol, and decreases in citrate, respectively. Gene expression analyses revealed increased expression of ATP-dependent citrate lyase (ACLY) and subsequent fatty acid biosynthetic enzymes, suggesting that OHT-treated MCF-7 cells activate citrate-to-lipid metabolism. The significance of the observed metabolic changes was evaluated by co-treating MCF-7 cells with OHT and ACLY or a diacylglycerol *O*-acyltransferase 1 (DGAT1) inhibitor. Co-treatment ameliorated cell death and reduced mitochondrial membrane potential compared to that in OHT treatment alone. The inhibition of cell death by co-treatment with an ACLY inhibitor has been observed in other breast cancer cell lines. These results suggest that citrate-to-lipid metabolism is critical for OHT-induced cell death in breast cancer cell lines.

## Introduction

Breast cancer is the most common cancer in women^1^. Breast cancer cells are classified based on the presence of hormone receptors, and 70% of these cells express the estrogen receptor (ER)^2^. Tamoxifen is a nonsteroidal estrogen receptor antagonist widely used in patients with breast cancer because of its low physical burden^3–5^. The active form of tamoxifen, 4-hydroxytamoxifen (OHT), is produced by CYP2D6 in the liver and induces growth arrest and apoptosis via competitive inhibition of estrogen receptors^6,7^. OHT is often used to investigate the direct role of tamoxifen in breast cancer in vitro experiments^8–10^.

Tamoxifen is known to cause various effects on cells^11^, including inhibition of the mitochondrial respiratory chain leading to loss of membrane potential^12^, inhibition of glucosylceramide synthase^13^, promotion of membrane localization of protein kinase C ε via the activation of phospholipases C and D^14,15^, and inhibition of cAMP phosphodiesterase by binding to calmodulin^16^. Tamoxifen affects ER-mediated apoptosis signal induction and various metabolic processes associated with the tricarboxylic acid (TCA) cycle and lipid metabolism, responsible for energy production and intracellular signaling^17,18^. Therefore, elucidating detailed metabolic responses will provide insights into the apoptotic effects of tamoxifen. Moreover, a holistic view of the metabolic alterations induced by OHT will further contribute to our understanding of its underlying mechanisms of action. Metabolomics and lipidomics using mass spectrometry are effective methods for studying metabolism because they allow the profiling of a wide range of metabolites and lipids^19,20^. Time-series metabolome and lipidome analyses can estimate metabolic changes from snapshots of the metabolite/lipid levels at each time point. In this study, time-series metabolomic and lipidomic analyses were performed on MCF-7 cells treated with OHT to elucidate the association between metabolic alterations and cell death. The identified metabolic alterations and their impact on cell death were validated using gene expression analysis and metabolic inhibitor co-treatment, respectively. The significance of these metabolic changes was also validated in various breast cancer cell lines.

## Results

### Untargeted lipidomics reveals upregulated lipogenesis in OHT-treated MCF-7 cells

**Figure 1:**
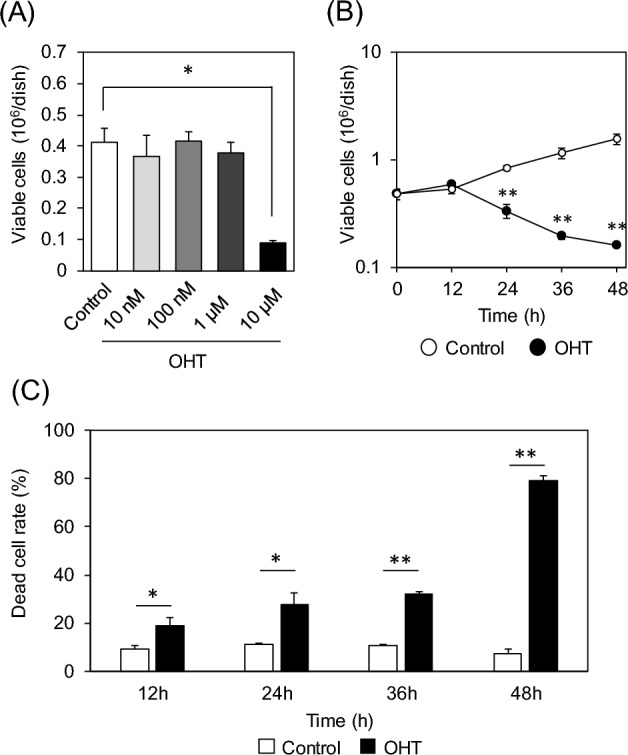
OHT-induced growth inhibition and cell death in MCF-7 cells. (**A**) OHT concentration-dependent growth inhibition after treatment for 48 h. (**B**) Proliferation curves of MCF-7 cells treated with 10 µM OHT. (**C**) Rates of dead MCF-7 cells treated with 10 µM OHT measured using a flow-cytometry-based apoptosis assay. All data are represented as means ± standard deviation (*n* = 3). *, adjusted *p* < 0.05; **, adjusted *p* < 0.01 in multiple Student’s *t*-test with Benjamini and Hochberg correction (FDR < 0.05).

**Figure 2:**
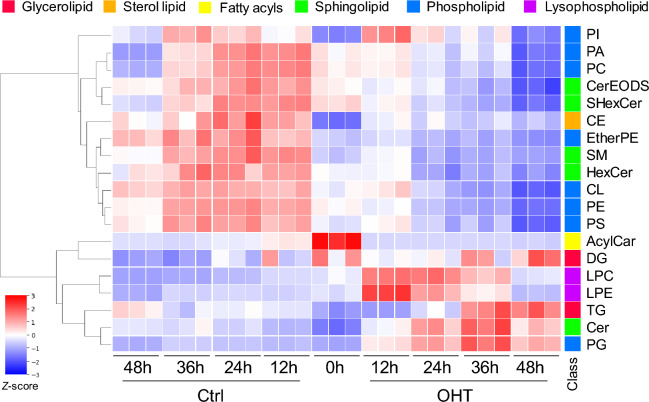
Lipid level alterations induced by OHT treatment in MCF-7 cells. The *Z*-score of the total intensity of each lipid class was subjected to hierarchical clustering and represented in a heatmap. PA, phosphatidic acid; PC, phosphatidylcholine; PE, phosphatidylethanolamine; PG, phosphatidylglycerol; PI, phosphatidylinositol; CL, cardiolipin; LPC, lysophosphatidylcholine; LPE, lysophosphatidylethanolamine; DG, diacylglycerol; TG, triacylglycerol; Cer, ceramide; SM, sphingomyelin; HexCer, hexosylceramide; SHexCer, sulfatide; CerEODS, Hexosylceramide esterified omega-hydroxy fatty acid-dihydrosphingosine; CE, cholesteryl ester; AcylCar, Acylcarnitine.

To investigate the association between metabolic changes and cell death induced by tamoxifen, OHT, the active form of tamoxifen, was added in MCF-7 cell medium. A significant decrease in the number of viable cells was observed upon 10 µM OHT treatment for 48 h (Fig. 1A). Time-course observations showed a gradual decrease in viable cell count after 12 h of treatment (Fig. 1B). A flow cytometry-based apoptosis assay revealed a time-dependent increase in the number of dead cells, reaching 79% after 48 h (Fig. 1C). Since previous studies have suggested that ceramide and phospholipid metabolism are altered in OHT-treated cells^11^, untargeted lipidomics has been performed to obtain a global perspective of lipid metabolism alterations. Single-phase extracts of whole-cell lipids were analyzed in data-dependent acquisition mode for LC-QTOF/MS, and 335 lipid molecular species were annotated. The score plots for the OHT treatment groups were separated from those of the control group, indicating that lipid metabolism was altered, according to principal component analysis (Fig. S1). Hierarchical clustering of the total intensities of each lipid subclass revealed that the levels of almost all phospholipids, except for phosphatidylglycerol, decreased, whereas those of diacylglycerol (DG) increased (Fig. 2). This is consistent with the results of a previous study showing that OHT treatment activates phospholipase C^14^. The respective increases and decreases in ceramide and hexosylceramide levels (Fig. 2) indicated that tamoxifen inhibited glucosylceramide synthase^13,18^. Interestingly, total triacylglycerol (TG) levels increased after 36–48 h of OHT treatment (Fig. 2). These data suggested that the activation of lipogenesis, in addition to the known changes in lipid metabolism reported in previous studies, occurred in OHT-treated MCF-7 cells.

### Metabolome analysis reveals a significant decrease in citrate levels in OHT-treated cells

**Figure 3:**
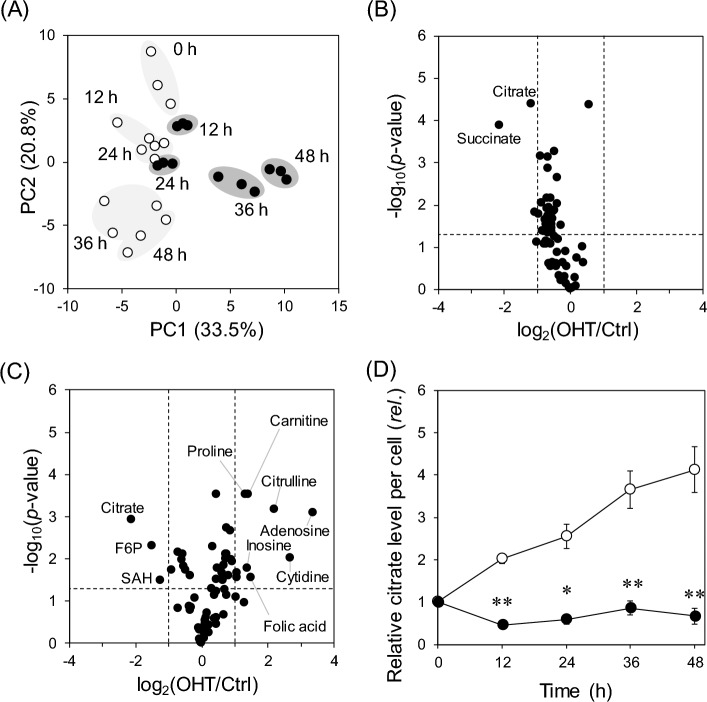
OHT-mediated alteration in central carbon metabolism in MCF-7 cells. (**A**) A principal component analysis of metabolome data. White and black circles indicate control and OHT treatment groups, respectively, with treatment time shown. (**B** and **C**) Volcano plots of metabolome data after (**B**) 12 and (**C**) 24 h treatment with OHT. Vertical dashed lines represent 2- and 0.5-fold changes. A horizontal dashed line represents a *p*-value of 0.05 via the Student's *t*-test. (**D**) Relative citrate levels charted over time. Data are represented as means ± standard deviation (*n* = 3). *, adjusted *p* < 0.05; **, adjusted *p* < 0.01 in multiple Student’s *t*-test with Benjamini and Hochberg correction (FDR < 0.05).

A metabolome analysis was performed to obtain more detailed insights into the effects of OHT on metabolism. A total of 62 polar metabolites were measured, including 21 amino acids and organic acids using GC–MS and 41 nucleic acids, sugar phosphates, vitamins, and amino acid derivatives using LC–MS/MS. Principal component analysis revealed that 33.5% and 20.8% of the total variation were explained by PC1 and PC2, respectively (Fig. 3A). The score plots for the OHT-treated groups deviated from those of the control group along PC1, indicating a change in metabolism after OHT treatment (Fig. 3A). Hierarchical clustering revealed decreased amino acid and sugar-phosphate levels in the OHT-treated group after 48 h (Fig. S2). To examine the metabolic alterations associated with the early stage of cell death, volcano plots of the metabolome data were created after 12 and 24 h of treatment (Fig. 3B,C). These plots demonstrate that the level of the common metabolite, citrate, decreased significantly (> twofold at 12 h and 24 h). The relative citrate level per cell remained consistently low from the early to the late stages in OHT-treated MCF-7 cells (Fig. 3D), suggesting an alteration in the metabolic pathways of citrate synthesis and degradation.

### Expression of genes involved in citrate synthesis and degradation is altered by OHT treatment

**Figure 4:**
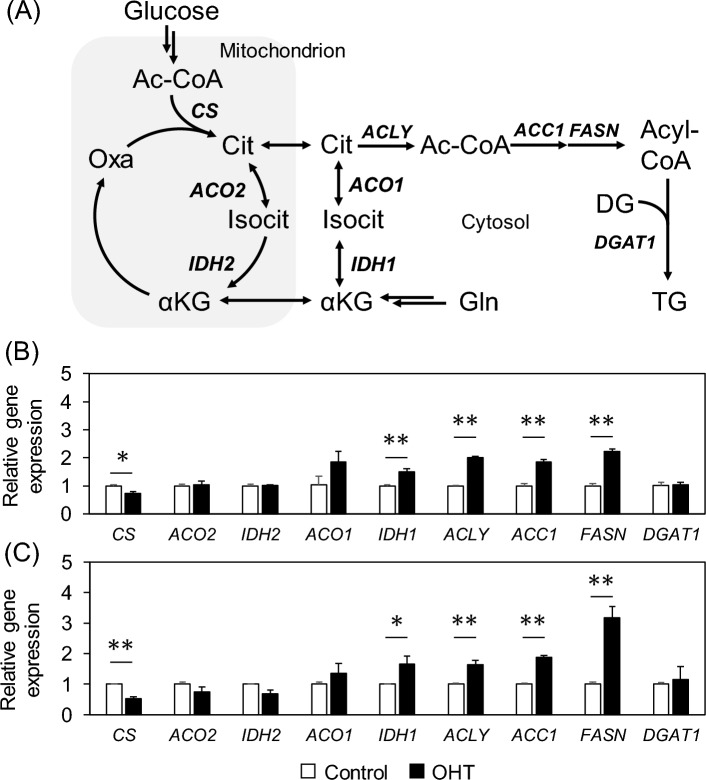
Effects of OHT treatment on metabolic enzyme gene expression levels. (**A**) A metabolic pathway of citrate synthesis and degradation. The grayed area represents a mitochondrion. (**B** and **C**) Relative gene expression levels of metabolic enzymes (**B**) 12 and (**C**) 24 h after OHT treatment. Data are represented as means ± standard deviation (*n* = 3). *, adjusted *p* < 0.05; **, adjusted *p* < 0.01 in multiple Student’s *t*-test with Benjamini and Hochberg correction (FDR < 0.05). Ac-CoA, acetyl-coenzyme A; Cit, citrate; Isocit, isocitrate; αKG, alpha-ketoglutarate; oxa, oxaloacetate.

To investigate the causes of decreased citrate levels induced by OHT treatment, the expression levels of citrate synthesis and degradation pathway genes were examined via quantitative PCR (Fig. 4A). Citrate synthase (*CS*) mRNA levels decreased to 73% and 52% at 12 and 24 h, respectively (Fig. 4B,C). No significant changes in gene expression were observed for aconitase (*ACO2*) and isocitrate dehydrogenase (*IDH2*), which metabolize citrate in the mitochondria (Fig. 4B,C). In contrast, the expression of ATP citrate lyase (*ACLY*), which cleaves citrate to acetyl-coenzyme A (Ac-CoA) in the cytosol, increased 2.0- and 1.6-fold at 12 and 24 h, respectively. Gene expression levels of the enzymes that synthesize fatty acids from cytosolic Ac-CoA (*ACC1* and *FASN*) significantly increased in a time-dependent manner (Fig. 4B,C), consistent with the data showing that lipid levels, such as TG, DG, and ceramides, were increased by OHT treatment (Fig. 2). Changes in *DGAT1* expression levels were not significant, probably because of post-translational regulation such as phosphorylation^21^. To determine the carbon source for lipid synthesis, the rates of specific glucose and glutamine uptake and lactate excretion were examined by medium component analysis (see Materials and Methods). Glutamine uptake increased with OHT treatment, whereas glucose uptake and lactate excretion rates remained constant from 12 to 48 h (Table S1). The metabolic fate of glutamine was examined using [U-^13^C_5_]glutamine. M + 3 and M + 4 labeling of malate, aspartate, and fumarate, which can be produced via the ACLY reaction, are indicators of reductive or oxidative glutamine metabolism, respectively (Fig. S3A,B). OHT treatment for 48 h increased M + 3 labeling, whereas it decreased M + 4 labeling (Fig. S3C–H), suggesting metabolic rewiring from oxidative to reductive glutamine metabolism^22^. This is consistent with a previous study showing that complex I is inhibited by OHT treatment^12^. These data suggested that the decrease in citrate levels observed in OHT-treated cells was due to the activation of lipid biosynthesis, which was fueled by reductive glutamine metabolism.

### ACLY and DGAT1 inhibition ameliorate OHT-induced cell death in MCF-7 cells

**Figure 5:**
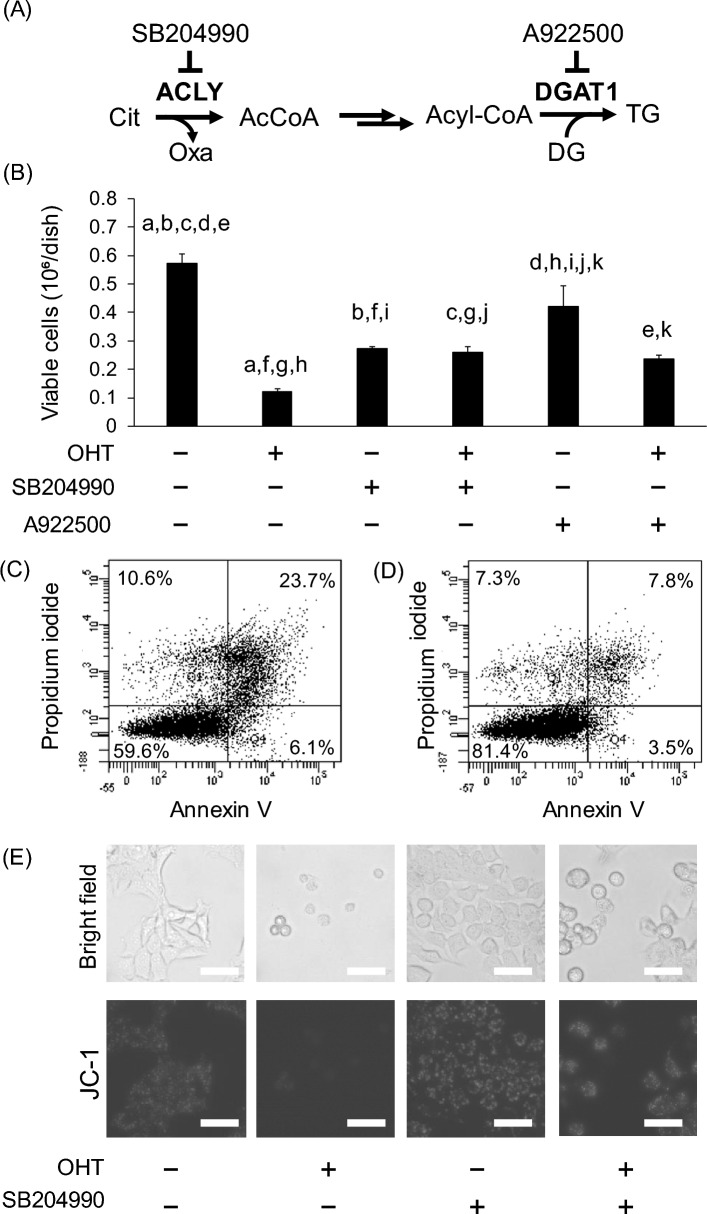
Suppression of OHT-induced cell death by inhibiting lipogenesis. (**A**) Schematic representation of the lipogenic pathway and inhibitors. (**B**) Viable MCF-7 cell numbers under OHT and ACLY inhibitor SB204990 or DGAT1 inhibitor A922599 treatment at 48 h. Data are represented as means ± standard deviation (*n* = 3). The same letters indicate* p* < 0.05 in one-way ANOVA by Tukey’s test. (**C** and **D**) Flow cytometric analysis of OHT-induced apoptotic cells with (**C**) DMSO and (**D**) SB204990 treatment. (**E**) Fluorescent microscopic analysis of mitochondria membrane potential with OHT and ACLY inhibitor SB204990 treatment using a JC-1 MitoMP detection kit. Upper row, bright field; lower row, fluorescent image. Scale bar, 50 µm.

To investigate the significance of OHT-induced citrate-to-lipid metabolism, cell proliferation was examined after treatment with ACLY and DGAT1 inhibitors (Fig. 5A). The decrease in the number of viable cells after 48 h of OHT treatment was alleviated by co-treating cells with OHT and the ACLY inhibitor SB204990 (60 µM) (Fig. 5B). This co-treatment increased the relative citrate levels (Fig. S4). Co-treatment with OHT and the DGAT1 inhibitor A922500 (50 µM) partly ameliorated the suppression of viable cell counts by OHT treatment. However, this change was not significant (Fig. 5B, *p* = 0.067, one-way ANOVA by Tukey’s test). An apoptosis assay using flow cytometry showed that co-treatment with OHT and SB204990 reduced the percentage of dead cells compared to that in OHT alone (Fig. 5C,D), indicating that the increase in viable cells with SB204990 co-treatment was due to the inhibition of apoptosis and necrosis. Consistent with this result, microscopic observation of mitochondrial membrane potential using the fluorescent reagent JC-1 revealed that mitochondrial membrane potential was lost in the OHT-alone treatment group and was maintained in the group co-treated with OHT and SB204990 (Fig. 5E). Similar results were obtained after co-treatment with OHT and A922500 (Fig. S5). These data suggest that the activation of metabolism from citrate degradation to TG synthesis is required for appropriate cell death in OHT-induced MCF-7 cells.

**Figure 6:**
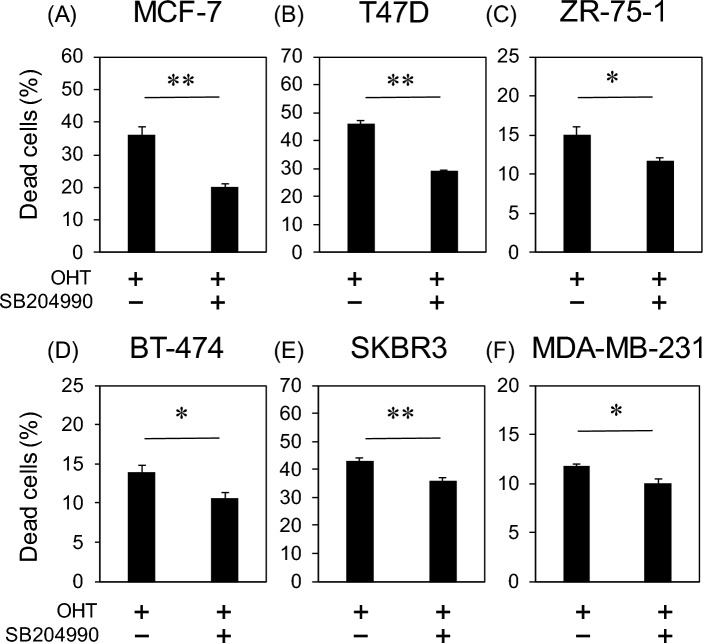
Dead cell rates in various OHT-treated breast cancer cell lines. The dead cell counts of (**A**) MCF-7, (**B**) T47D, (**C**) ZR-75–1, (**D**) BT-474, and (**E**) MDA-MB-231 cells were determined using flow cytometry. Data are represented as means ± standard deviation (*n* = 3). *,* p* < 0.05, **,* p* < 0.01 in the Student’s *t*-test.

The generality of this phenomenon was evaluated in other cell lines. Breast cancer cell lines were classified into luminal (MCF-7, T47D, and ZR-75-1), HER2 (SKBR3), and triple-negative (MDA-MB-231) subtypes. These cells were treated with 15 µM OHT, with or without 60 µM SB204990, and rates of dead cells were measured using flow cytometry. In luminal cell lines, including MCF-7 and T47D cells, co-treatment with SB204990 approximately halved the dead cell rate (Fig. 6A,B). Although the reduction in cell death rates by the co-treatment with OHT and SB204990 was small in other cell lines, the dead cell rates decreased in all cell lines (Fig. 6C–F). The limited reduction in dead cell rates was possibly due to relatively low OHT sensitivity. These results indicate that the inhibition of OHT-induced cell death by ACLY inhibitors is a common phenomenon in breast cancer cell lines.

## Discussion

In this study, we used lipidomic and metabolomic techniques to analyze OHT-induced metabolic alterations in MCF-7 cells. Untargeted lipidomics revealed an increase in DG and TG levels, along with known alterations in ceramide and phospholipid metabolism (Fig. 2). Metabolome analysis revealed that citrate levels decreased during the early stage (12–24 h) of OHT treatment (Fig. 3B,C). Although OHT is metabolized and detoxified by cytochrome P450^23^, the continuous increase in dead cells suggests that the observed metabolic changes were not due to recovery from the effects of OHT (Fig. 1B,C). Consistent with the metabolic alterations, quantitative PCR showed a decrease and increase in the gene expression levels of enzymes involved in citrate biosynthesis and degradation, respectively, and an upregulation of the gene expression of fatty acid biosynthetic enzymes, ACC1 and FASN, following OHT treatment (Fig. 4B,C). The upregulation of lipogenic enzyme expression is believed to contribute to the increase in TG, DG, and ceramide levels (Fig. 2). As these lipids are associated with apoptosis^24–26^, the significance of the metabolic changes induced by OHT was evaluated by adding an ACLY inhibitor to OHT-mediated cell death. ACLY catalyzes a key reaction linking citrate and lipid metabolism. The addition of the ACLY inhibitor SB204490 suppressed OHT-induced apoptosis (Fig. 5), indicating that ACLY-mediated citrate withdrawal for lipid metabolism is critical for the cell death induced by OHT.

Tamoxifen has been shown to induce apoptosis via a decrease in mitochondrial membrane potential, attributed to the inhibition of respiratory chain complex I^12^. The complex I inhibitor rotenone significantly decreases citrate levels in HeLa cells^27^. Based on these previous studies, the decrease in citrate levels observed after OHT treatment is believed to be mediated by complex I inhibition. This study adds to the detailed observation that citrate levels can be reduced by upregulating the citrate degradation pathway toward lipogenesis while downregulating citrate synthesis (Fig. 4). Citrate withdrawal is likely to rapidly stagnate mitochondrial metabolism, resulting in a loss of membrane potential. ACLY inhibition possibly contributes to the prevention of citrate withdrawal, thus maintaining mitochondrial membrane potential. The increase in citrate levels after ACLY inhibition supported this hypothesis (Fig. S4). Previous studies demonstrated that tamoxifen is effective against estrogen receptor-negative breast cancer cell lines^11^. Our findings revealed that the inhibitory effect of ACLY on OHT-induced cell death occurred in the estrogen receptor-negative breast cancer cell lines SKBR3 and MDA-MB-231 (Fig. 6). This suggests that citrate withdrawal for lipid synthesis is likely involved in the estrogen receptor-independent effects of tamoxifen.

ACLY is a key enzyme that catalyzes the starting point for fatty acid synthesis, and it is a potential target for anti-cancer drugs because previous studies have shown that ACLY inhibition dramatically inhibits cell proliferation^28,29^. Consistent with this, a single treatment with the ACLY inhibitor, SB204490, reduced the number of viable cells (Fig. 5B). In contrast, in this study, co-treatment with ACLY inhibitors suppressed OHT-induced cell death (Fig. 5B). Since the accumulation of lipid droplets in apoptotic cells has been widely observed^30,31^, the activation of ACLY and citrate withdrawal for lipogenesis appear to be common phenomena in cell death. Further research is needed to determine whether ACLY inhibition similarly inhibits cell death induced by other anti-cancer agents.

## Methods

### Cell lines, media, and culture

The breast cancer cell lines MCF-7, T47D, ZR-75-1, BT-474, SKBR3, and MDA-MB-231 were obtained from RIKEN Bioresource Research Center (Tsukuba, Japan). The cells were maintained in phenol red-free Dulbecco’s modified Eagle’s medium (DMEM) (Fujifilm Wako Pure Chemical Corporation, Osaka, Japan) containing 10% fetal bovine serum (Life Technologies, Carlsbad, CA, USA) and 1% penicillin/streptomycin (Fujifilm Wako Pure Chemical Corporation) at 37 °C with 5% CO_2_. For time-series metabolomics, 3.5 × 10^5^ cells were seeded in 3.5 mL of medium in 60-mm-diameter dishes. After a 15-h incubation, following rapid washing twice with FBS-free media, cells were cultured in 3.5 mL DMEM lacking glucose, l-glutamine, phenol red, sodium pyruvate, and sodium bicarbonate (Sigma-Aldrich, St. Louis, MO, USA), supplemented with 20 mM glucose, 2.0 mM glutamine, 3.7 g/L sodium bicarbonate, and 10% dialyzed FBS (Life Technologies, Carlsbad, CA, USA). [U-^13^C_5_] Glutamine was used for isotope tracing experiments. The cells were treated with (*Z*)-4-hydroxytamoxifen (Sigma-Aldrich, St. Louis, MO, USA) in ethanol and SB204990 (MedChemExpress, Monmouth Junction, NJ, USA) or A922599 (MedChemExpress) in dimethyl sulfoxide (DMSO). In all experiments, control cell cultures were treated with an equivalent volume of the corresponding solvent.

### Extraction of metabolites and lipids

Following rapid aspiration of culture medium and washing with 1 mL of cold phosphate buffered saline (PBS), cellular metabolism was quickly quenched by adding 800 µL of ice-cold methanol containing 5 µM norvaline and 10-camphor sulfonic acid (internal standards) as described previously^32^. Cells were collected by scraping and stored at − 80 °C until extraction. Metabolites were extracted using bilayer extraction as described previously^32^. Briefly, 800 µL of chloroform and 320 µL of Milli-Q water were added and mixed for 1 min each with vortexing and sonication. After centrifugation at 2580 × *g* for 20 min at 4 °C and incubation for 10 min, 600 µL of the upper phase was dispensed into three 1.5-mL microcentrifuge tubes (200 µL each), and 800 µL of the lower phase was evaporated to dryness in a 2-mL glass tube using a centrifugal evaporator (CVE-2100, EYELA). All samples were stored at − 80 °C until analysis.

### GC–MS analysis for polar metabolites

The dried residue of the upper phase was derivatized via methoxyamination and *tert*-butyldimethylsilylation and analyzed using GC–MS in selected ion mode, as described previously^33^. Briefly, dried residue obtained from the upper phase was dissolved in 50 µL of 40 mg/mL methoxyamine hydrochloride in pyridine and incubated for 1 h at 30 °C, then 50 µL of *N*-methyl-*N*-(*tert*-butyldimethylsilyl)trifluoroacetamide containing 1% *tert*-butyldimethylchlorosilane was added and incubated for 1 h at 60 °C. After cooling for over 2 h, the supernatant was analyzed using gas chromatography/mass spectrometry (GCMS-QP2020, Shimadzu, Kyoto, Japan) with a DB-5MS + DG capillary column (30 m, 0.25 mm, 0.25 µm, Agilent Technologies, Santa Clara, CA, USA) in selected ion monitoring mode. Analysis conditions were as follows: carrier gas, helium; flow rate, 1.0 mL/min; inlet temperature, 250 °C; injection volume, 1 µL; injection mode, split (10:1); oven temperature gradient: 60 °C for 3.5 min, increased at a rate of 10 °C/min to 325 °C; ionization; electron ionization at 70 eV; ion source temperature, 200 °C; interface temperature, 250 °C.

### LC-tripleQ/MS analysis for polar metabolites

The dried residue of the upper phase was dissolved in 50 µL of Milli-Q water and centrifuged at 6000 × *g* for 5 min. The supernatant was analyzed using two different liquid chromatography-triple quadrupole mass spectrometry (LC-tripleQ/MS) systems in the multiple reaction monitoring mode. A Nexera X2 (Shimadzu) equipped with MASTRO C18 (150 mm, 2.1 mm, 3 µm, Shimadzu GLC, Tokyo, Japan) coupled to LCMS-8050 (Shimadzu) was operated using the methods package for primary metabolites Ver. 2 (Shimadzu). The settings were as follows: solvent A, Milli-Q water containing 15 mM tributylamine and 10 mM acetic acid; solvent B, methanol; gradient, 0 min, 0% (B); 0.5 min, 0% (B); 8 min, 25% (B); 12 min, 98% (B); 15 min, 98% (B); 15.1 min, 0% (B); 20 min, 0% (B); flow rate, 0.3 mL/min; injection volume, 1 µL; polarity, negative; ionization, ESI; nebulizer gas flow, 3 L/min; heating gas flow, 10 L/min; interface temperature, 300 °C; DL temperature, 250 °C; heat block temperature, 400 °C; drying gas flow, 10 L/min.

The same samples were analyzed using a Nexera X2 (Shimadzu) equipped with Discovery HS F5 (150 mm, 2.1 mm, 3 µm, Sigma-Aldrich) coupled to LCMS-8040 (Shimadzu)^34^. The mobile phases consisted of (A) ultrapure water (Fujifilm Wako Pure Chemical Corporation) containing 0.1% formic acid (Fujifilm Wako Pure Chemical Corporation) and (B) acetonitrile (Fujifilm Wako Pure Chemical Corporation). The metabolites were eluted at a flow rate of 0.25 mL/min and column temperature of 40 °C with the following gradient: 0 min, 0% (B); 5 min, 0% (B); 15 min, 40% (B); 15.1 min, 100% (B); 18 min, 100% (B); 18.1 min, 0%; and 25 min, 0% (B). MS settings are as follows: ionization mode, ESI; nebulizer gas, 3.0 L/min; drying gas, 10.0 L/min; DL temperature, 250 °C; and heat block temperature, 400 °C.

### LC-QTOF/MS analysis for lipids

The dried residue of the lower phase was dissolved in 100 µL of chloroform containing 1 µL of Equisplash (Avanti polar lipids, Birmingham, AL, USA). After centrifugation at 6000 × *g* for 5 min, 50 µL of this supernatant was transferred to a vial. Lipids were analyzed using a Nexela X2 equipped with ACQUITY UPLC Peptide BEH C18 (50 mm, 2.1 mm, 1.7 µm, Waters, Milford, MA, USA) coupled to a quadrupole time-of-flight (QTOF)/MS (LCMS-9030, Shimadzu) operated in data-dependent acquisition mode as described previously^35,36^. MS-DIAL ver. 3.98 was used to identify lipid molecule species^37^.

### Analysis of metabolite uptake and secretion rates

The rates of glucose and glutamine uptake and lactate secretion were determined by the increase or decrease in their concentrations in the culture medium and the number of viable cells. Glucose and lactate concentrations were measured using a Prominence (Shimadzu) equipped with a refractive index detector and an Aminex HPX-87H column (Bio-Rad)^32^. The mobile phase was a 1.5 mM H_2_SO_4_ solution. The flow rate and column temperature were 0.5 mL/min and 65 °C, respectively. The glutamine levels were measured using the AccQ-Tag method^38^. Specific uptake and secretion rates (*ρ*) were calculated following equation^39^:

$$P = \rho \mathop \smallint \limits_{t1}^{t2} X\left( t \right)dt + P_{0}$$

### Data analysis

Peak areas obtained using metabolome and lipidome analyses were divided by viable cell numbers and peak areas of internal standards and then normalized by calculating the *Z*-score. Principal component analysis and hierarchical clustering were performed in Python 3.7.6 using Scikit-learn ver. 0.22.1, Seaborn ver. 01.10.0, and Pandas 1.0.1.

### Apoptosis assay

Apoptotic cells were identified using an Annexin V-FITC/PI apoptosis detection kit (Nacalai Tesque, Kyoto, Japan), following the manufacturer’s protocol. Cells were washed with PBS and resuspended in 100 µL of Annexin V binding solution, followed by adding 5 µL of Annexin V-FITC and 5 µL of propidium iodide and incubation for 15 min at room temperature. Flow cytometry was performed using a BD FACSAria IIIu cell sorter (BD Biosciences, San Jose, CA, USA) after stained cells were washed and resuspended in 500 µL of Annexin V binding solution.

### RNA extraction and real-time PCR

Total RNA was isolated using a NucleoSpin® RNA kit (Takara Bio Inc., Shiga, Japan) according to the manufacturer’s protocol. Complementary DNA was synthesized from 250 ng of RNA using PrimeScript™ RT Master Mix (Perfect Real Time, Takara Bio Inc.). Real-time PCR was performed using a StepOne Plus Real-Time PCR System (Applied Biosystems, Waltham, MA, USA) and TB Green® Premix Ex Taq® II (Tli RNase H Plus, Takara Bio Inc.). Target gene sequences were amplified using the forward and reverse primers listed in Table S2 after initial denaturation at 95 °C for 30 s, followed by 40 cycles of 95 °C for 5 s and annealing and elongation at 60 °C for 30 s. Sequences encoding glyceraldehyde 3-phosphate dehydrogenase (GAPDH) were used as an internal control. Relative gene expression levels were calculated using the ΔΔCt method.

### Supplementary Information

Supplementary Information.

## Data Availability

All data was included in the main text and supplementary files.
