# Supplementary material for: Lipidome and metabolome analyses reveal metabolic alterations associated with MCF-7 apoptosis upon 4-hydroxytamoxifen treatment
Source: Sci Rep. 2023 Oct 29;13:18549. doi: 10.1038/s41598-023-45764-2 (PMC10613619; doi:10.1038/s41598-023-45764-2)
Supplement: Supplementary file 1 — Supplementary Information. [file 41598_2023_45764_MOESM1_ESM.docx]

**Supplementary information**

**Title**

Lipidome and metabolome analyses reveal metabolic alterations associated with MCF-7 cell apoptosis upon 4-hydroxytamoxifen treatment.

**Authors**

Kazuki Nishimoto^1^, Nobuyuki Okahashi^1,2^, Masaharu Maruyama^1^, Yoshihiro Izumi^3^, Kohta Nakatani^3^, Yuki Ito^2,4^, Junko Iida^2,4^, Takeshi Bamba^3^ and Fumio Matsuda^1,2,^*

**Affiliations**

^1^Department of Bioinformatic Engineering, Graduate School of Information Science and Technology, Osaka University, 1-5 Yamadaoka, Suita, Osaka 565-0871, Japan

^2^Department of Biotechnology, Osaka University Shimadzu Analytical Innovation Research Laboratory, Graduate School of Engineering, Osaka University, 2-1 Yamadaoka, Suita 565-0871, Japan

^3^Division of Metabolomics, Medical Research Center for High Depth Omics, Medical Institute of Bioregulation, Kyushu University, 3-1-1 Maidashi, Higashi-ku, Fukuoka 812-8582, Japan

^4^Analytical and Measuring Instruments Division, Shimadzu Corporation, 1 Nishinokyo Kuwabara-cho, Nakagyo-ku, Kyoto 604-8511, Japan

**Supplementary tables**

**Table S1 Specific rates**

| Specific rates | Control | OHT treatment |
| --- | --- | --- |
| Glucose uptake (nmol/10^6^ cells/h) | 678 ± 31 | 636 ± 76 |
| Glutamine uptake (nmol/10^6^ cells/h) | 113 ± 6 | 370 ± 21 |
| Lactate secretion (nmol/10^6^ cells/h) | 1591 ± 65 | 1596 ± 114 |

**Table S2 Primer sequences used in quantitative PCR**

| Target genes | Forward or Reverse | Sequences |
| --- | --- | --- |
| *ACC1* | Forward | GCTTATTGATCAGTTGTGTGGCC |
| *ACC1* | Reverse | GCGTGCTCGAAGTGCTAC |
| *ACO1* | Forward | GCAGGCACCACAGACTATCC |
| *ACO1* | Reverse | CTGCACCGTACTCTTTGCCAG |
| *ACO2*^1^ | Forward | CCAGAGACCGACTACCTGACG |
| *ACO2*^1^ | Reverse | CCACTTGTCAAAAGGCTCCAG |
| *ACLY*^2^ | Forward | GAAGGGAGTGACCATCATCG |
| *ACLY*^2^ | Reverse | TTAAAGCACCCAGGCTTGAT |
| *CS*^3^ | Forward | CGGAAGTTCCTGGAGCACCTCTC |
| *CS*^3^ | Reverse | AAGTACACCTTGGCCCCCACGTA |
| *FASN*^4^ | Forward | CTTCCGAGATTCCATCCTACGC |
| *FASN*^4^ | Reverse | TGGCAGTCAGGCTCACAAACG |
| *GAPDH*^4^ | Forward | ATGGAAATCCCATCACCATCTT |
| *GAPDH*^4^ | Reverse | CGCCCCACTTGATTTTGG |
| *IDH1* | Forward | GTATGAGCATAGGCTCATCGACG |
| *IDH1* | Reverse | CATGCCGAGAGAGCCATACC |
| *IDH2* | Forward | ATGGTGGCTCAGGTCCTCAAG |
| *IDH2* | Reverse | GCCTCAGCCTCAATCGTCTTCC |

**Supplementary figures**

 **Figure S1. Principal component analysis of lipidome data**

White and black circles indicate control and OHT treatment groups, respectively, labeled with treatment times.

**Figure S2. Hierarchical clustering of metabolome data**

*Z*-scores of the peak intensity were subjected to hierarchical clustering and presented as a heatmap.

**Figure S3. A tracer experiment using [U-^13^C_5_]glutamine**

(A and B) Atom mapping of (A) reductive and (B) oxidative glutamine metabolism. White and black circles represent 12C and 13C atoms, respectively.

(C-H) ^13^C-labeling ratio of M+3 (C) aspartate, (D) fumarate, and (E) malate, and M+4 (F) aspartate, (G) fumarate, and (H) malate at 48 hours. Effects of naturally occurring isotopes were corrected^5^.

Data are represented as means±standard deviation (*n* = 3). *, *p* <0.05; **, *p* <0.01 in Student’s *t*-test.

**Figure S4. Relative citrate level in cells co-treated with OHT and ACLY inhibitor**

Data are presented as means±standard deviation (*n* = 3). *, adjusted *p* < 0.05, **, adjusted *p* < 0.01 in multiple Student’s *t*-test with Benjamini and Hochberg correction (FDR<0.05).

**Figure S5.** **Fluorescent microscopic image of mitochondrial membrane potential**

Cells were treated with OHT and the DGAT1 inhibitor A922599. Upper row, bright field images; lower row, fluorescence images. Scale bar, 50 µm.

**Supplementary references**

1. Fodor, T. *et al.* Combined Treatment of MCF-7 Cells with AICAR and Methotrexate, Arrests Cell Cycle and Reverses Warburg Metabolism through AMP-Activated Protein Kinase (AMPK) and FOXO1. *PLoS One* **11**, e0150232 (2016).

2. Wang, D., Yin, L., Wei, J., Yang, Z. & Jiang, G. ATP citrate lyase is increased in human breast cancer, depletion of which promotes apoptosis. *Tumor Biol.* **39**, (2017).

3. Peng, M. *et al.* Intracellular citrate accumulation by oxidized ATM-mediated metabolism reprogramming via PFKP and CS enhances hypoxic breast cancer cell invasion and metastasis. *Cell Death Dis.* **10**, (2019).

4. Li, J. *et al.* Fatty Acid Synthase Mediates the Epithelial-Mesenchymal Transition of Breast Cancer Cells. *Int. J. Biol. Sci.* **10**, 171 (2014).

5. Okahashi, N., Yamada, Y., Iida, J. & Matsuda, F. Isotope Calculation Gadgets : A Series of Software for Isotope-Tracing Experiments in Garuda Platform. *Metabolites* **12**, (2022).
